# Hexaaqua­copper(II) bis­(tetra­fluorido­borate)–pyrazine 1,4-dioxide (1/3)

**DOI:** 10.1107/S1600536813007629

**Published:** 2013-03-23

**Authors:** Jan L. Wikaira, Christopher P. Landee, Mark M. Turnbull

**Affiliations:** a Department of Chemistry, University of Canterbury, Private Bag 4800, Christchurch, New Zealand; b Department of Physics, Clark University, 950 Main St, Worcester, MA 01610, USA; c Department of Chemistry, Clark University, 950 Main St, Worcester, MA 01610, USA

## Abstract

The crystal structure of the title compound, [Cu(H_2_O)_6_](BF_4_)_2_·3C_4_H_4_N_2_O_2_, comprises discrete [Cu(H_2_O)_6_]^2+^ cations and BF_4_^−^ anions along with three equivalents of pyrazine 1,4-dioxide (pzdo). The hexa­aqua­copper(II) ion and all three pzdo mol­ecules lie about crystallographic inversion centers. The lattice is supported by an extensive hydrogen-bonding network. O—H⋯O hydrogen bonding between the [Cu(H_2_O)_6_]^2+^ and pzdo units creates a pseudo-hexa­gonal lattice parallel to the *bc* plane. The BF_4_^−^ anions lie in the voids of that lattice, held in place by O—H⋯F hydrogen bonds, and also generate BF_4_^−^–pzdo–BF_4_^−^–pzdo stacks *via* short F⋯N contacts [2.866 (3)–3.283 (4) Å].

## Related literature

For related structures see: Blake *et al.* (2000 ▶) [*catena*-(tris­(μ^3^-pyrazino­(2,3-*f*)quinoxaline)tris­ilver(I) tris­(tetra­fluorido­bor­ate) nitro­methane)]; Muesmann *et al.* (2011 ▶) [hexa­aqua­copper(II) 2,3,5,6-tetra­fluoro-1,4-benzene­disulfonate]; Jia *et al.* (2005 ▶) [hexa­kis­(tricyclo­hexyl­phosphine oxide) hexa­aqua­copper(II) bis­(tetra­fluoridoborate) sesquihydrate]; Ma *et al.* (2001 ▶) [hexa­aqua­copper(II) dichloride (4,4′-bipyridine-*N*,*N*′-dioxide) dihydrate]; Lu *et al.* (2009 ▶) [*N*,*N*′-diethyl­pyrazine­diium bis­(tetra­fluoridoborate)]; Turksoy *et al.* (2003 ▶) [2,5-bis­(2-meth­oxy­phen­yl)-3,6-dimethyl­pyrazinium bis­(tetra­fluorido­borate)]; Schlueter *et al.* (2012 ▶) [*catena*-[(μ^2^-pyrazine-*N*,*N*′-dioxide)diaqua­dichloro­copper(II)]; Shatruk *et al.* (2006 ▶) [Cu(II)(hat) tetra­fluoridoborate; hat = 1,4,5,8,9,12-hexa­aza­triphenyl­ene]; Verbitsky *et al.* (2008 ▶) [2,3-dicyano-1-ethyl-5-(4-fluoro­phen­yl)pyrazinium tetra­fluoridoborate].
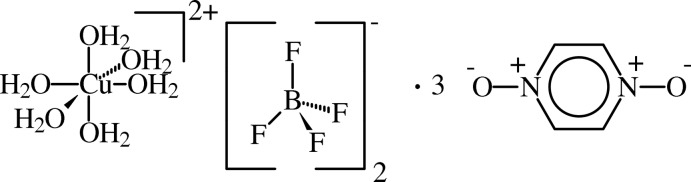


## Experimental

### 

#### Crystal data

[Cu(H_2_O)_6_](BF_4_)_2_·3C_4_H_4_N_2_O_2_
*M* *_r_* = 681.53
Triclinic, 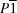

*a* = 6.4001 (4) Å
*b* = 10.2719 (7) Å
*c* = 10.9162 (8) Å
α = 110.928 (6)°
β = 104.327 (6)°
γ = 93.937 (5)°
*V* = 639.59 (7) Å^3^
*Z* = 1
Cu *K*α radiation
μ = 2.40 mm^−1^
*T* = 120 K
0.50 × 0.4 0× 0.35 mm

#### Data collection

Agilent SuperNova (Dual, Cu at zero, Atlas) diffractometer
Absorption correction: multi-scan (*CrysAlis PRO*; Agilent, 2011 ▶) *T*_min_ = 0.781, *T*_max_ = 1.000
3851 measured reflections
2399 independent reflections
2356 reflections with *I* > 2σ(*I*)
*R*_int_ = 0.015

#### Refinement

*R*[*F* ^2^ > 2σ(*F* ^2^)] = 0.045
*wR*(*F* ^2^) = 0.111
*S* = 1.06
2399 reflections
206 parameters
H atoms treated by a mixture of independent and constrained refinement
Δρ_max_ = 1.47 e Å^−3^
Δρ_min_ = −0.85 e Å^−3^

### 

Data collection: *CrysAlis PRO* (Agilent, 2011 ▶); cell refinement: *CrysAlis PRO*; data reduction: *CrysAlis PRO*; program(s) used to solve structure: *SHELXTL* (Sheldrick, 2008 ▶); program(s) used to refine structure: *SHELXTL*; molecular graphics: *SHELXTL* and *Mercury* (Macrae *et al.*, 2008 ▶); software used to prepare material for publication: *SHELXL97*, *enCIFer* (Allen *et al.*, 2004 ▶) and *publCIF* (Westrip, 2010 ▶).

## Supplementary Material

Crystal structure: contains datablock(s) I, global. DOI: 10.1107/S1600536813007629/sj5306sup1.cif

Structure factors: contains datablock(s) I. DOI: 10.1107/S1600536813007629/sj5306Isup2.hkl

Additional supplementary materials:  crystallographic information; 3D view; checkCIF report

## Figures and Tables

**Table 1:** Hydrogen-bond geometry (Å, °)

*D*—H⋯*A*	*D*—H	H⋯*A*	*D*⋯*A*	*D*—H⋯*A*
O1—H1*A*⋯O31^i^	0.80 (4)	1.93 (4)	2.711 (3)	166 (4)
O1—H1*B*⋯O21^ii^	0.76 (4)	1.94 (4)	2.676 (3)	166 (4)
O2—H2*A*⋯O31	0.81 (4)	1.93 (4)	2.735 (3)	173 (4)
O2—H2*B*⋯O11	0.77 (4)	1.89 (5)	2.669 (3)	179 (4)
O3—H3*A*⋯O11^i^	0.74 (4)	2.07 (4)	2.806 (3)	177 (4)
O3—H3*B*⋯F4^iii^	0.74 (4)	2.28 (4)	2.984 (4)	158 (4)
O3—H3*B*⋯F3^iii^	0.74 (4)	2.41 (4)	3.042 (3)	145 (4)

Symmetry codes: (i) 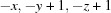; (ii) 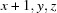; (iii) 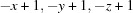.

**Fig. 1.**
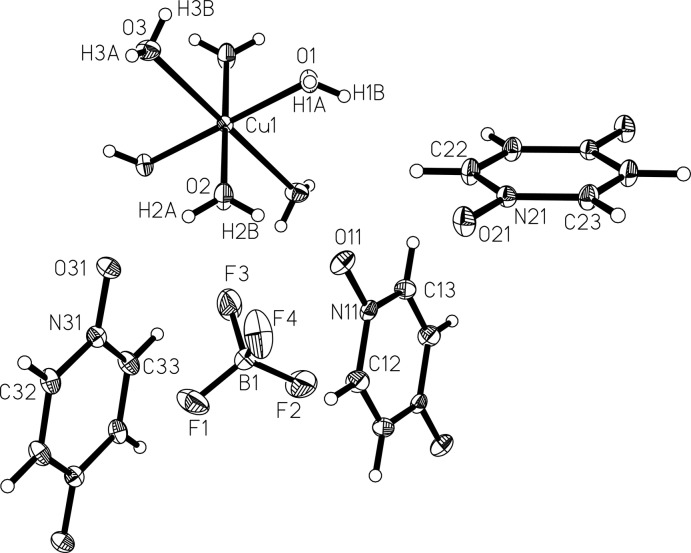
: Thermal ellipsoid plot of 1 (50% probability) showing the asymmetric unit and the three 1,4-pyrazinedioxide molecule that lie about inversion centers. Labeled atoms are related to unlabeled atoms by the symmetry operations: -x+1, -y+1, -z+1 for Cu(H2O)6,2+, -x, -y+1, -z for N11, C12 and C13; -1-x, -y, -z for N21, C22 and C23. Only those hydrogen atoms whose positions were refined and are involved in hydrogen-bonding are labeled.

**Fig. 2.**
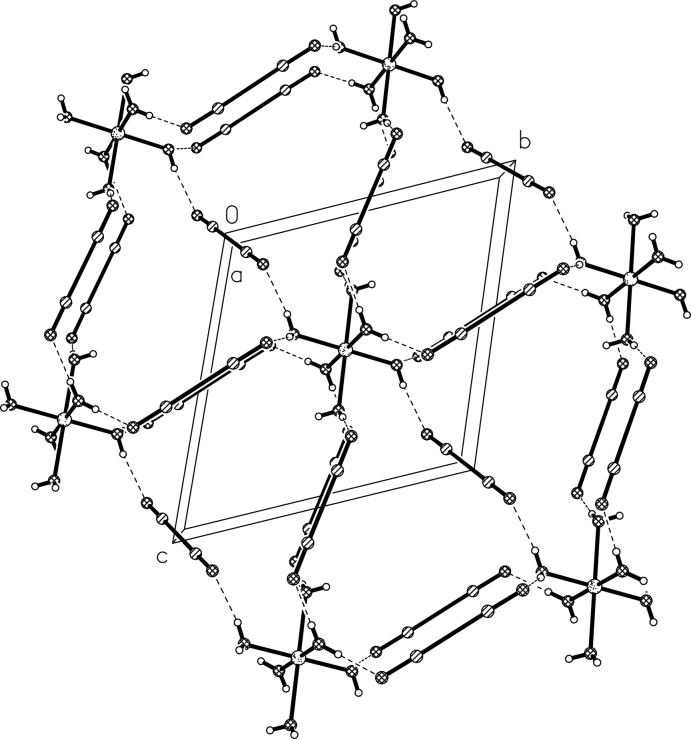
: Diagram showing hydrogen-bonded [Cu(H2O)6]2+ and pzdo moieties which generate a pseudo-hexagonal lattice parallel to the bc-plane. Pzdo molecules have been represented by only the N– and O-atoms, with a solid line linking the N-atoms within a single ring, for clarity. Dashed lines represent hydrogen bonds.

**Fig. 3.**
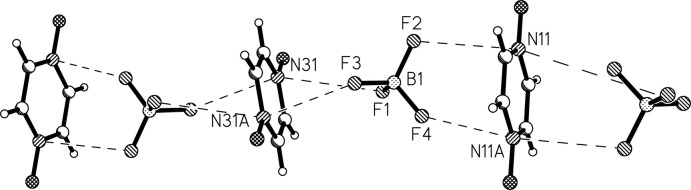
: Diagram showing the stacking of pzdo molecules and BF4- anions. Short F···N contacts are shown as dashed lines.

## supplementary crystallographic information

### Comment

Hexaaquacopper(II) tetrafluoroborate tris-1,4-pyrazinedioxide, 1, crystallized from aqueous solution as part of our efforts in the synthesis of low-dimensional Cu(II) antiferromagnetic lattices (Schlueter, *et al.* 2012). The hydroscopic material crystallizes as discrete [Cu(H_2_O)_6_]^2+^ cations with the Cu^2+^ atoms located on inversion centers and BF_4_^-^ anions along with three equivalents of 1,4-pyrazine dioxide (pzdo) each of which sits athwart an individual inversion center, Fig. 1. The lattice is supported by an extensive hydrogen bonding network between the coordinated water molecules and both the tetrafluoroborate anions and the pzdo molecules. The [Cu(H_2_O)_6_]^2+^ cations are linked, *via* hydrogen bonds with water molecules O1 and O2, into a pseudo-hexagonal layer parallel to the *bc*-plane *via* the pzdo molecules (see Figure 2). A similar compound with hexaaquacopper(II) chloride and 4,4'-bipyridine-N,*N*'-dioxide molecule (Ma *et al.*, 2001) also shows hydrogen bonding to the amine oxide O-atoms, but does not generate the same layer pattern, likely due to the much greater length of the ligand and smaller anion which would create large cavities in the structure. The BF_4_^-^ anions occupy the roughly triangular holes generated by that lattice and are held in place *via* hydrogen bonds to the O3 water molecules. Additional hydrogen bonds link the layers parallel to the *a*-axis, generating a three-dimensional structure.

Further structural support for the lattice is found *via* pairs of short contacts between the BF_4_^-^ anions and the pyrazine nitrogen atoms which generate a chain-like motif (Figure 3). These contact distances range in length from 2.866 (3) to 3.283 (4) Å. Similar F···N interactions are seen in a number of complexes with F···N distances near 3 Å, such as the Cu(II)(hat) tetrafluoroborate complex (Shatruk *et al.*, 2006), while distances as short as 2.837 Å have been reported in 2,5-bis(2-methoxyphenyl)-3,6-dimethylpyrazinium bis(tetrafluoroborate) (Turksoy *et al.*, 2003) and *catena*-(tris(µ3-pyrazino(2,3 - f) quinoxaline)-tri-silver(I) tris(tetrafluoroborate) nitromethane) (Blake *et al.*, 2000). Fewer structures are known with pairs of short F···N contacts to a single ring, such as seen in 1. These include 2,5-bis(2-methoxyphenyl)- 3,6-dimethylpyrazinium bis(tetrafluoroborate) (Turksoy *et al.*, 2003), 2,3-dicyano-1-ethyl-5-(4-fluorophenyl)pyrazinium tetrafluoroborate (Verbitsky *et al.*, 2008) and *N*,*N*'-diethylpyrazinediium bis(tetrafluoroborate) (Lu *et al.*, 2009). We find no examples of the stacked pairwise interactions resulting in a chain-like motif such as seen in 1.

### Experimental

Copper(II) tetrafluoroborate hexahydrate and 1,4-pyrazine dioxide were dissolved in equimolar amounts in water. The solution was left for slow evaporation in air at room temperature. Over the course of two months, crystals of compound 1 were recovered. The crystals are hygroscopic and were transferred directly from the mother liquor into a perfluoropolyalkylether for data collection.

### Refinement

All H-atoms bound to carbon were refined using a riding model with d(C—H) = 0.93 Å, *U*_iso_=1.2*U*_eq_ (C). Hydrogen atoms bonded to oxygen atoms were located in the difference map and their positions refined using fixed isotropic U values.

### Crystal data

**Table:**

[Cu(H_2_O)_6_](BF_4_)_2_·3C_4_H_4_N_2_O_2_	*Z* = 1
*M**_r_* = 681.53	*F*(000) = 345
Triclinic, *P*1	*D*_x_ = 1.769 Mg m^−^^3^
Hall symbol: -P 1	Cu *K*α radiation, λ = 1.54184 Å
*a* = 6.4001 (4) Å	Cell parameters from 3193 reflections
*b* = 10.2719 (7) Å	θ = 4.5–73.6°
*c* = 10.9162 (8) Å	µ = 2.40 mm^−^^1^
α = 110.928 (6)°	*T* = 120 K
β = 104.327 (6)°	Block, light blue
γ = 93.937 (5)°	0.5 × 0.4 × 0.35 mm
*V* = 639.59 (7) Å^3^	

### Data collection

**Table:**

Agilent SuperNova (Dual, Cu at zero, Atlas) diffractometer	2399 independent reflections
Radiation source: fine-focus sealed tube	2356 reflections with *I* > 2σ(*I*)
Graphite monochromator	*R*_int_ = 0.015
Detector resolution: 10.6501 pixels mm^-1^	θ_max_ = 70.1°, θ_min_ = 4.5°
ω scans	*h* = −7→7
Absorption correction: multi-scan (*CrysAlis PRO*; Agilent, 2011)	*k* = −8→12
*T*_min_ = 0.781, *T*_max_ = 1.000	*l* = −13→12
3851 measured reflections	

### Refinement

**Table:**

Refinement on *F*^2^	Secondary atom site location: difference Fourier map
Least-squares matrix: full	Hydrogen site location: difference Fourier map
*R*[*F*^2^ > 2σ(*F*^2^)] = 0.045	H atoms treated by a mixture of independent and constrained refinement
*wR*(*F*^2^) = 0.111	*w* = 1/[σ^2^(*F*_o_^2^) + (0.0476*P*)^2^ + 1.833*P*] where *P* = (*F*_o_^2^ + 2*F*_c_^2^)/3
*S* = 1.06	(Δ/σ)_max_ = 0.001
2399 reflections	Δρ_max_ = 1.47 e Å^−^^3^
206 parameters	Δρ_min_ = −0.85 e Å^−^^3^
0 restraints	Extinction correction: *SHELXTL* (Sheldrick, 2008), Fc^*^=kFc[1+0.001xFc^2^λ^3^/sin(2θ)]^-1/4^
Primary atom site location: structure-invariant direct methods	Extinction coefficient: 0.0258 (19)

### Special details

**Table:**

Geometry. All e.s.d.'s (except the e.s.d. in the dihedral angle between two l.s. planes) are estimated using the full covariance matrix. The cell e.s.d.'s are taken into account individually in the estimation of e.s.d.'s in distances, angles and torsion angles; correlations between e.s.d.'s in cell parameters are only used when they are defined by crystal symmetry. An approximate (isotropic) treatment of cell e.s.d.'s is used for estimating e.s.d.'s involving l.s. planes.
Refinement. Refinement of *F*^2^ against ALL reflections. The weighted *R*-factor *wR* and goodness of fit *S* are based on *F*^2^, conventional *R*-factors *R* are based on *F*, with *F* set to zero for negative *F*^2^. The threshold expression of *F*^2^ > σ(*F*^2^) is used only for calculating *R*-factors(gt) *etc*. and is not relevant to the choice of reflections for refinement. *R*-factors based on *F*^2^ are statistically about twice as large as those based on *F*, and *R*- factors based on ALL data will be even larger.

### Fractional atomic coordinates and isotropic or equivalent isotropic displacement parameters (Å^2^)

**Table:**

	*x*	*y*	*z*	*U*_iso_*/*U*_eq_	
Cu1	0.5000	0.5000	0.5000	0.0101 (2)	
O1	0.3542 (3)	0.3009 (2)	0.4022 (2)	0.0147 (4)	
H1A	0.234 (7)	0.289 (4)	0.409 (4)	0.018*	
H1B	0.340 (6)	0.266 (4)	0.326 (4)	0.018*	
O2	0.2162 (3)	0.5606 (2)	0.4493 (2)	0.0226 (5)	
H2A	0.178 (6)	0.631 (5)	0.493 (4)	0.027*	
H2B	0.131 (7)	0.525 (4)	0.378 (5)	0.027*	
O3	0.4517 (4)	0.5081 (2)	0.7053 (2)	0.0184 (4)	
H3A	0.355 (7)	0.525 (4)	0.729 (4)	0.022*	
H3B	0.475 (6)	0.439 (4)	0.709 (4)	0.022*	
N11	−0.0418 (4)	0.4676 (2)	0.1034 (2)	0.0140 (5)	
O11	−0.0811 (3)	0.4372 (2)	0.2040 (2)	0.0196 (4)	
C12	−0.1425 (5)	0.5635 (3)	0.0635 (3)	0.0189 (6)	
H12	−0.2414	0.6076	0.1069	0.023*	
C13	0.1006 (5)	0.4040 (3)	0.0397 (3)	0.0179 (6)	
H13	0.1705	0.3378	0.0661	0.022*	
N21	−0.6322 (4)	0.0764 (2)	0.0681 (2)	0.0120 (5)	
O21	−0.7602 (3)	0.1492 (2)	0.1319 (2)	0.0173 (4)	
C22	−0.4126 (4)	0.1135 (3)	0.1184 (3)	0.0165 (6)	
H22	−0.3508	0.1915	0.2000	0.020*	
C23	−0.7198 (4)	−0.0376 (3)	−0.0509 (3)	0.0149 (5)	
H23	−0.8710	−0.0643	−0.0866	0.018*	
N31	0.0263 (4)	0.8928 (2)	0.5452 (2)	0.0136 (5)	
O31	0.0475 (3)	0.7870 (2)	0.5868 (2)	0.0171 (4)	
C32	−0.1066 (4)	0.9842 (3)	0.5873 (3)	0.0159 (6)	
H32	−0.1798	0.9747	0.6481	0.019*	
C33	0.1346 (4)	0.9090 (3)	0.4589 (3)	0.0167 (6)	
H33	0.2287	0.8472	0.4306	0.020*	
B1	0.4841 (5)	0.8023 (4)	0.2195 (3)	0.0183 (6)	
F1	0.4718 (4)	0.9441 (2)	0.2745 (2)	0.0390 (5)	
F2	0.4757 (4)	0.7527 (3)	0.0834 (2)	0.0472 (6)	
F3	0.6823 (4)	0.7802 (2)	0.2911 (2)	0.0506 (7)	
F4	0.3235 (4)	0.7182 (3)	0.2381 (3)	0.0523 (7)	

### Atomic displacement parameters (Å^2^)

**Table:**

	*U*^11^	*U*^22^	*U*^33^	*U*^12^	*U*^13^	*U*^23^
Cu1	0.0087 (3)	0.0093 (3)	0.0117 (3)	0.00147 (19)	0.0027 (2)	0.0033 (2)
O1	0.0142 (10)	0.0137 (9)	0.0148 (10)	−0.0002 (7)	0.0067 (8)	0.0028 (8)
O2	0.0159 (10)	0.0231 (11)	0.0179 (11)	0.0095 (9)	−0.0011 (8)	−0.0016 (9)
O3	0.0209 (11)	0.0207 (11)	0.0208 (10)	0.0065 (9)	0.0114 (8)	0.0122 (9)
N11	0.0117 (10)	0.0164 (11)	0.0105 (11)	0.0015 (8)	0.0007 (8)	0.0031 (9)
O11	0.0179 (10)	0.0281 (11)	0.0145 (10)	0.0035 (8)	0.0048 (8)	0.0102 (8)
C12	0.0163 (13)	0.0230 (14)	0.0188 (14)	0.0097 (11)	0.0068 (11)	0.0076 (12)
C13	0.0158 (13)	0.0182 (14)	0.0186 (14)	0.0072 (11)	0.0026 (11)	0.0064 (11)
N21	0.0109 (10)	0.0121 (10)	0.0125 (11)	0.0031 (8)	0.0041 (8)	0.0037 (9)
O21	0.0123 (9)	0.0180 (10)	0.0181 (10)	0.0059 (7)	0.0069 (7)	0.0010 (8)
C22	0.0134 (13)	0.0162 (13)	0.0141 (13)	0.0010 (10)	0.0020 (10)	0.0008 (10)
C23	0.0100 (12)	0.0163 (13)	0.0142 (13)	0.0007 (10)	0.0012 (10)	0.0028 (10)
N31	0.0110 (10)	0.0122 (10)	0.0157 (11)	−0.0003 (8)	0.0031 (9)	0.0041 (9)
O31	0.0174 (10)	0.0139 (9)	0.0222 (10)	0.0030 (7)	0.0074 (8)	0.0084 (8)
C32	0.0141 (13)	0.0147 (13)	0.0177 (13)	0.0013 (10)	0.0079 (10)	0.0030 (11)
C33	0.0148 (13)	0.0151 (13)	0.0220 (14)	0.0048 (10)	0.0107 (11)	0.0053 (11)
B1	0.0147 (15)	0.0237 (16)	0.0178 (15)	0.0024 (12)	0.0063 (12)	0.0088 (13)
F1	0.0526 (13)	0.0337 (11)	0.0487 (13)	0.0253 (10)	0.0301 (11)	0.0230 (10)
F2	0.0762 (17)	0.0527 (14)	0.0303 (11)	0.0296 (13)	0.0287 (11)	0.0243 (10)
F3	0.0430 (13)	0.0381 (12)	0.0410 (13)	0.0166 (10)	−0.0132 (10)	−0.0035 (10)
F4	0.0483 (14)	0.0424 (13)	0.0538 (15)	−0.0145 (11)	0.0319 (12)	−0.0031 (11)

### Geometric parameters (Å, º)

**Table:**

Cu1—O1	1.9692 (19)	N21—O21	1.307 (3)
Cu1—O1^i^	1.9692 (19)	N21—C22	1.348 (4)
Cu1—O2	1.974 (2)	N21—C23	1.355 (3)
Cu1—O2^i^	1.974 (2)	C22—C23^iii^	1.363 (4)
Cu1—O3^i^	2.310 (2)	C22—H22	0.9300
Cu1—O3	2.310 (2)	C23—C22^iii^	1.363 (4)
O1—H1A	0.80 (4)	C23—H23	0.9300
O1—H1B	0.76 (4)	N31—O31	1.322 (3)
O2—H2A	0.81 (4)	N31—C32	1.346 (4)
O2—H2B	0.77 (4)	N31—C33	1.348 (4)
O3—H3A	0.74 (4)	C32—C33^iv^	1.366 (4)
O3—H3B	0.74 (4)	C32—H32	0.9300
N11—O11	1.318 (3)	C33—C32^iv^	1.366 (4)
N11—C13	1.346 (4)	C33—H33	0.9300
N11—C12	1.353 (4)	B1—F2	1.373 (4)
C12—C13^ii^	1.363 (4)	B1—F1	1.379 (4)
C12—H12	0.9300	B1—F3	1.395 (4)
C13—C12^ii^	1.363 (4)	B1—F4	1.397 (4)
C13—H13	0.9300		
			
O1—Cu1—O1^i^	180.00 (14)	C13^ii^—C12—H12	119.7
O1—Cu1—O2	89.66 (9)	N11—C13—C12^ii^	120.1 (3)
O1^i^—Cu1—O2	90.34 (9)	N11—C13—H13	119.9
O1—Cu1—O2^i^	90.34 (9)	C12^ii^—C13—H13	119.9
O1^i^—Cu1—O2^i^	89.66 (9)	O21—N21—C22	121.0 (2)
O2—Cu1—O2^i^	180.00 (15)	O21—N21—C23	120.0 (2)
O1—Cu1—O3^i^	87.37 (8)	C22—N21—C23	119.0 (2)
O1^i^—Cu1—O3^i^	92.63 (8)	N21—C22—C23^iii^	120.7 (3)
O2—Cu1—O3^i^	88.50 (9)	N21—C22—H22	119.7
O2^i^—Cu1—O3^i^	91.50 (9)	C23^iii^—C22—H22	119.7
O1—Cu1—O3	92.63 (8)	N21—C23—C22^iii^	120.4 (2)
O1^i^—Cu1—O3	87.37 (8)	N21—C23—H23	119.8
O2—Cu1—O3	91.50 (9)	C22^iii^—C23—H23	119.8
O2^i^—Cu1—O3	88.50 (9)	O31—N31—C32	120.1 (2)
O3^i^—Cu1—O3	180.000 (1)	O31—N31—C33	120.5 (2)
Cu1—O1—H1A	112 (3)	C32—N31—C33	119.3 (2)
Cu1—O1—H1B	118 (3)	N31—C32—C33^iv^	120.4 (3)
H1A—O1—H1B	104 (4)	N31—C32—H32	119.8
Cu1—O2—H2A	127 (3)	C33^iv^—C32—H32	119.8
Cu1—O2—H2B	123 (3)	N31—C33—C32^iv^	120.2 (3)
H2A—O2—H2B	109 (4)	N31—C33—H33	119.9
Cu1—O3—H3A	127 (3)	C32^iv^—C33—H33	119.9
Cu1—O3—H3B	105 (3)	F2—B1—F1	113.8 (3)
H3A—O3—H3B	109 (4)	F2—B1—F3	107.4 (3)
O11—N11—C13	120.2 (2)	F1—B1—F3	108.8 (3)
O11—N11—C12	120.6 (2)	F2—B1—F4	109.6 (3)
C13—N11—C12	119.2 (2)	F1—B1—F4	111.9 (3)
N11—C12—C13^ii^	120.7 (3)	F3—B1—F4	104.7 (3)
N11—C12—H12	119.7		
			
O11—N11—C12—C13^ii^	179.4 (2)	O21—N21—C23—C22^iii^	179.5 (2)
C13—N11—C12—C13^ii^	−0.2 (5)	C22—N21—C23—C22^iii^	0.0 (4)
O11—N11—C13—C12^ii^	−179.4 (2)	O31—N31—C32—C33^iv^	−177.9 (2)
C12—N11—C13—C12^ii^	0.2 (4)	C33—N31—C32—C33^iv^	1.1 (4)
O21—N21—C22—C23^iii^	−179.5 (2)	O31—N31—C33—C32^iv^	177.9 (2)
C23—N21—C22—C23^iii^	0.0 (4)	C32—N31—C33—C32^iv^	−1.1 (4)

Symmetry codes: (i) −*x*+1, −*y*+1, −*z*+1; (ii) −*x*, −*y*+1, −*z*; (iii) −*x*−1, −*y*, −*z*; (iv) −*x*, −*y*+2, −*z*+1.

### Hydrogen-bond geometry (Å, º)

**Table:**

*D*—H···*A*	*D*—H	H···*A*	*D*···*A*	*D*—H···*A*
O1—H1*A*···O31^v^	0.80 (4)	1.93 (4)	2.711 (3)	166 (4)
O1—H1*B*···O21^vi^	0.76 (4)	1.94 (4)	2.676 (3)	166 (4)
O2—H2*A*···O31	0.81 (4)	1.93 (4)	2.735 (3)	173 (4)
O2—H2*B*···O11	0.77 (4)	1.89 (5)	2.669 (3)	179 (4)
O3—H3*A*···O11^v^	0.74 (4)	2.07 (4)	2.806 (3)	177 (4)
O3—H3*B*···F4^i^	0.74 (4)	2.28 (4)	2.984 (4)	158 (4)
O3—H3*B*···F3^i^	0.74 (4)	2.41 (4)	3.042 (3)	145 (4)

Symmetry codes: (i) −*x*+1, −*y*+1, −*z*+1; (v) −*x*, −*y*+1, −*z*+1; (vi) *x*+1, *y*, *z*.
